# Supplementary material for: Social capital and cognitive decline: Does sleep duration mediate the association?
Source: PLoS One. 2021 May 27;16(5):e0252208. doi: 10.1371/journal.pone.0252208 (PMC8158899; doi:10.1371/journal.pone.0252208)
Supplement: Attachment — Submitted filename: Response to Reviewers.docx [file pone.0252208.s002.docx]

Dear editor:

We are submitting the revised manuscript titled “Social capital and cognitive decline: does sleep duration mediate the association?”. We have modified the manuscript along the lines suggested by editors and reviewers，now, it is more precise and more robust.

Below are our responses to the reviewer’s comments point by point.

**Editor comments**

1. In agreement with suggestions of Reviewer 2 I feel that the analysis, and possibly the message, of the paper should undergo a really major revision, as present conclusions seem to have limited support from data.

**Response:** We have modified the manuscript entirely according to reviewer’s suggestion; now we believe it better than the previous version.

2. In fact the main message of the paper is that social capital is associated with the risk of cognitive decline. Yet correlations between measures of SC and MMSE are small. Social cohesion has a correlation of .12, while social interaction has a correlation of 0.09. Moreover, based on Table 3, it looks like social cohesion is not associated with MMSE in univariate analysis.

**Response:** Because the research on social capital is biased towards sociology, there are many potential influencing factors, and the effect size is small (usually the effect value of social science is small, but it does not affect the existence of the effect), and this study mainly to explore the mediation effect. In the multivariate analysis, there is a weak correlation between social capital and cognitive decline. The path analysis shows that sleep duration played a mediator role in the relationship between social capital and cognitive decline.

3. The whole hypothesis of the mediation is made uncertain by the cross-sectional design. In fact both low social capital and longer sleep duration might be consequence, rather than predictors, of cognitive decline. In fact the present message of the paper would be that older subjects should sleep less to maintain their social capital, that seems at odds with evidence suggesting an association between shorter sleep time and increased risk of cognitive decline.

**Response:** We have discussed our findings more comprehensively; now, it’s easier for the reader to understand the relationship between social capital, sleep duration, and cognitive decline. In this study, we mainly focus on the mediation effect of sleep duration, and the results showed social capital was positively associated with cognitive function and negatively associated with sleep duration; meanwhile, sleep duration was also negatively associated with cognitive function, so long sleep duration might be a risk factor for cognitive function. Furthermore, a longitudinal cohort study [1] reported possessing a rich social capital has been associated with better cognitive function among the old adults. Robbins et al. found lower social capital members in groups were seen for long sleepers [2]. Additionally, as described in a review [3], sleep fragmentation can result in poor quality sleep, depression, and underlying disease processes, such as CHD, which appear to be relevant to the association between long sleep and cognitive function. Reverse causality, the possibility that cognitive function determines sleep duration, cannot be ruled out in our analyses. However, the longitudinal data [4] revealed that sleep duration “increase from 7 or 8 hours” was associated with lower cognitive function scores. Meanwhile, a previous study [5] has also provided no firm evidence that cognitive decline predicts sleep duration.

[1]. [Bennett DA](https://www.ncbi.nlm.nih.gov/pubmed/?term=Bennett%20DA%5BAuthor%5D&cauthor=true&cauthor_uid=16632311), [Schneider JA](https://www.ncbi.nlm.nih.gov/pubmed/?term=Schneider%20JA%5BAuthor%5D&cauthor=true&cauthor_uid=16632311), [Tang Y](https://www.ncbi.nlm.nih.gov/pubmed/?term=Tang%20Y%5BAuthor%5D&cauthor=true&cauthor_uid=16632311), [Arnold SE](https://www.ncbi.nlm.nih.gov/pubmed/?term=Arnold%20SE%5BAuthor%5D&cauthor=true&cauthor_uid=16632311), [Wilson RS](https://www.ncbi.nlm.nih.gov/pubmed/?term=Wilson%20RS%5BAuthor%5D&cauthor=true&cauthor_uid=16632311). The effect of social networks on the relation between Alzheimer's disease pathology and level of cognitive function in old people: a longitudinal cohort study. [Lancet Neurol.](https://www.ncbi.nlm.nih.gov/pubmed/?term=The+effect+of+social+networks+on+the+relation+between+Alzheimer%27s+disease+pathology+and+level+of+cognitive+function+in+old+people%3A+a+longitudinal+cohort+study) 2006; 5(5): 406-412.

[2] Robbins R , Jean-Louis G , Gallagher R A , et al. Examining Social Capital in Relation to Sleep Duration, Insomnia, and Daytime Sleepiness[J]. Sleep Medicine, 2019.

[3] Grandner MA, Drummond SP. Who are the long sleepers? Towards an understanding of the mortality relationship. Sleep Med Rev 2007;11:341-60.

[4] Ferrie J E , Shipley M J , Akbaraly T N , et al. Change in sleep duration and cognitive function: findings from the Whitehall II Study.[J]. Sleep(5):565-73.

[5] Yaffe K, Blackwell T, Barnes DE, Ancoli-Israel S, Stone KL. Preclinical cognitive decline and subsequent sleep disturbance in older women. Neurology 2007;69:237-42.

4. As further statistical suggestion, MMSE distribution is probably non-normal. It would be probably better to categorize it and perform a multinomial regression instead of a linear regression analysis. It would be of interest to include in the same model as predictors social capital, sleep time and interaction between the two factors.

**Response:** Yes, as you mentioned, the MMSE score fit a non-normal distribution. Thus, we performed a logarithmic transformation of the MMSE score to finish the mediation effect modeling that requires continuous data, and linear regression employed in the univariate analysis consequently. Besides, categorizing continuous variables may lose much information of the original variable and severely downward precision of the effect estimation.

**Reviewer #1**

1. Introduction: Overlay, this section has been prepared orderly. In the last paragraph, authors want to point the relationship between sleep duration and cognitive functions and properly reference to the study which conclude that homocysteine and beta amyloid may mediate the effects of poor sleep on cognition. However, ref 22 (smoking and sleep) here is not related to the main issue and I think it should be removed and instead another relevant ref can be added.

**Response:** Now, we have removed the description of “previous study revealed that sleep duration plays a significant mediating role in the relationship between smoking and mild cognitive impairment [22]” in the last paragraph of introduction and ref 22 (smoking and sleep).

2. Line 133: the fifth domain of MMSE is vague. Please refine it.

**Response:** We has refined the fifth domain of MMSE as constructional praxis (coping task, eg. copying intersecting pentagons), can be seen in line 132 -133.

3. Sleep duration: I am wondering why the authors did not consider the sleep quality rather than sleep duration. Then, they could easily use the well-known and standard scale, Pittsburgh sleep questionnaire

**Response:** The relationship between sleep quality and health has been well studied in the past decades; We believe sleep duration is one dimension of sleep quality, at least among old adults. And more and more research has focused on the relationship between sleep duration and cognitive function; to our knowledge, no study was conducted among Chinses old adults who were living in different cultural backgrounds from other countries.

4. Discussion: in the second paragraph which deals with the association between sleep duration and cognition, authors remark the sleep fragmentation and OSA as the possible causations of long sleep duration and low MMSE scales. But, I can’t understand why the authors mention here to the short sleep duration as this is a very definite and old finding and also they have no data on this issue. Rather, more relevant findings regarding the physiological alteration associated with long sleep duration could be of most benefits.

**Response:** We removed the description of short sleep duration. And there another possible explanation: “Besides, long sleep duration has been associated with an increased level of inflammatory factors and elevated inflammatory cytokine levels increase the risk of cognitive decline” in line 240.

5. Discussion: the last paragraph regarding the positive correlation of SC and MMSE scores could be discussed more deeply with mentioning to the some relevant basic findings.

**Response:** We have extensively edited the discussion section to explain the relationship between SC and cognitive function. See “Furthermore, a recent study manifested that social capital could help older adults continue to independently live in local communities and handle life stressors efficiently, even when they encounter declines in their physical and cognitive health. Additionally, social capital can provide older residents with a sense of security and belonging and is an important reserve domain in old age [54].”

6. The whole manuscript should be proof read for some grammatical and spelling errors. The example is in Line 210: there no!

**Response:** We have checked the grammatical and spelling errors entirely by our colleague at Duke University. All the changes are marked in red.

**Reviewer #2**

1. There are several issues with the overall writing style and appropriate use of grammar. This issue extends throughout the article.

**Response:** We have checked the grammatical and spelling errors entirely by our colleague at Duke University. All the changes are marked in red.

2. On line 125, “MMSE” is used for the first time in the text of the article, though the acronym is not explained until the following paragraph. Acronyms should be spelled out upon their first use. This made understanding line 125 a bit difficult.

**Response:** Many thanks for your suggestions. We have spelled out the acronym of MMSE in the last third line of the field process part and marked in red.

3. Further, the explanation of how the MMSE is scored in the Method section is a bit tricky to decipher. It is unclear if lower scores translate to cognitive impairment, or if higher scores do. This will help with understanding the beginning of the discussion section where the results are summarized.

**Response:** We have clarified this concern in line 132 in the Methods section; the higher MMSE score reflects the lower cognitive decline levels.

4. On line 194, no r values are given for the association between social cohesion/interaction and MMSE. Only one p-value is provided here.

**Response:** We have added r values and p-value for the association between social cohesion/interaction and MMSE. See line 195 “both the social cohesion (r=0.12, P<0.001) and social interaction (r=0.09, P<0.05) was positively correlated with MMSE score”.

5. On line 221, the author states “SC leads to better cognitive performance…” However, this study cannot provide any causal interpretations of the data since there was no manipulation of variables.

**Response:** We have rewritten the sentence as “the positive relationship between SC and better cognitive performance in older Chinese adults was found”, can be seen in line 224-225.

6. On line 222, the author states that SC is negatively associated with risk of cognitive decline, but the correlations between social cohesion/interaction and MMSE were positive. Further, on line 251, the author then states that SC is positively correlated with MMSE. These statements should be edited so that they agree with the data.

**Response:** As we noted in line 132, a higher MMSE score reflects the lower cognitive decline levels. So SC is positively correlated with MMSE means, which is negatively associated with the risk of cognitive decline.

7. In the results, it was unclear why analyses were done both with those who slept less than 8 hours and those who slept 8 hours +. Perhaps a brief discussion of this in the methods or in the results section would help the reader better understand this analysis.

**Response:** The average sleep duration of the total participants was 8.1 hours, and we categorized the sleep duration into two groups using a cutoff point of 8 hours. We revised this as “Considering the U-shape relationship between sleep duration and cognitive decline, the exploring analysis conducted stratified (as a cutoff point of mean sleep duration of the total population) by sleep duration showed in Table 5”.

8. The paragraph starting on line 226 seems to be making the point that there is evidence that both long and short sleep are connected to cognitive decline. However, the language pointing to this conclusion seems to be a little vague in this paragraph. Specifically, there is a sudden jump from talking about evidence about longer sleep duration to shorter sleep duration. A transition word or sentence might help to bridge this gap and clarify the overall point of this paragraph.

**Response:** Same with the editor’s comments 3, now, we have removed the description of short sleep duration.

9. The paragraph starting on line 242 seems to have a similar issue. The point here appears to be that people with more social capital are busier, and thus are getting less sleep, but this point again seems to be vague and somewhat difficult for the reader to decipher.

**Response:** Due to the U-shape relationship between sleep duration and cognitive function, here our findings support that people with high social activities may keep them in appropriate sleep duration, that means not too short neither too long. Another possible explanation as you mentioned that keep busy may helpful for older adults stay in bed unexpected long time.

10. The measurement of sleep duration appears to be problematic. Specifically, I’m not sure how appropriate it is to simply add 1 hour to the overall calculated sleep duration if someone answers “yes” to the question about whether they napped. Ideally, a follow-up question could have asked respondents who indicated napping how long they were asleep during the nap. This kind of information is fairly easy to record, so it is unclear why that was not originally built into the design.

**Response:**  We have mentioned in the limitation part as “bedtime and sleep duration were collected via a self-reported survey question; it may involve information bias despite it be commonly used in the epidemiological study due to the feasibility consideration”. In addition, the previous research [1] reported a good correlation when comparing the measurement (subjective and actigraphic measurement) of sleep timing and duration. Furthermore, here the bedtime we asked was the actual sleep onset time, and considering the local life culture, people may like an afternoon nap; we also ask the question ‘Do you sleep at the afternoon?’.

55. Lockley S W, Skene D J, Arendt J. Comparison between subjective and actigraphic measurement of sleep and sleep rhythms. Journal of Sleep Research. 1999; 8:175-183.

11. The measurement of alcohol use seems a bit unclear. Based on the article, it appears that this might have been a yes/no measurement (i.e., they either were or were not an alcohol user). However, the question asks if they’ve had one drink in the last 12 months. If this is the case, it seems as though this question is missing out on quite a bit of variation in use of alcohol. I’m also not sure if it would be appropriate to classify someone who had one drink nearly a year ago as an alcohol user.

**Response:** Yes, we have defined the alcohol use as: a drink at least one glass of alcohol (that equal to 1/2 bottle of beer or 125-milliliter grape wine or fruit wine or 40-milliliter white wine) in the past 12 months, seen in line 165-167. In our study, alcohol users include someone who had one drink a year ago, and in the past year.

12. In the results, the author does not mention the fact that correlations between measures of SC and MMSE are small. Social cohesion has a correlation of .12, while social interaction has a correlation of .009. While these may be significant, they are quite small. Recognition of this fact might be appropriate to discuss in the results and discussion sections.

**Response:** Because the research on social capital is biased towards sociology, there are many potential influencing factors, and the effect size is small (usually the effect value of social science is small, but it does not affect the existence of the effect), and this study mainly explores the mediation effect. In the multivariate analysis, there is a weak correlation between social capital and cognition. The analysis shows that sleep duration is the mediation variable. The interpretability of the analysis results is explained from the perspective of path analysis. We supplemented the description in line 196-197 of results and marked in red.

13. Based on Table 3, it looks like social cohesion is not associated with MMSE under model 1, but that it is under model 2. However, in the text, the authors say that social cohesion is associated with MMSE for model 1, and that this association remains after controlling for covariates under model 2. Any results given in-text and in tables/diagrams should match.

**Response:** We have revised the description of the tables, and now the results given in text matched the tables.

14. Further, because social capital was measured in two ways (i.e., social cohesion and social interaction), results for these two measures might be easier to understand if they are separated in the text. Perhaps focusing on the analysis for cohesion first and then interaction under separate headings would allow the reader to understand the results for both of these measures more easily.

**Response:** We have clarified this as: Social capital is measured with one instrument with two dimensions, social cohesion and social interaction, not two ways, so we analyzed together in our study.
