# Supplementary material for: Social capital and cognitive decline: Does sleep duration mediate the association?
Source: PLoS One. 2021 May 27;16(5):e0252208. doi: 10.1371/journal.pone.0252208 (PMC8158899; doi:10.1371/journal.pone.0252208)
Supplement: Attachment — Submitted filename: Response to Reviewers.docx [file pone.0252208.s003.docx]

Dear editor:

We are submitting the revised manuscript titled “Social capital and cognitive decline: does sleep duration mediate the association?” for publication. We have modified the manuscript along the lines suggested by editors and reviewers. All the changes are marked in red.

Below are our responses to the reviewer’s comments point by point.

ACADEMIC EDITOR:

1. I have appreciated the way the Authors have refocuesed the discussion. However I would advise to be more cautious whan (in the Abstract) thay state that "improvement of sleep duration may help in maintaining cognitive function", as literally this would be to make people sleep less, which is clearly not the message of the data.

**Response:** We have rephrased the conclusion as " It may be a suggestive clue to identify those at a higher risk of progressing to cognitive impairment.”

2. Moreover, I have asked for a statistical revision of the manuscript, as I was not sure of the methodological robustness of findings, due to the small association observed between social cohesion and cognitive function (even non significant in Regression model 1, Table 3), thus making the whole assumptions of a mediation analysis uncertain.

**Response:** Our results found a significant association between social cohesion and cognitive function after adjust the potential covriates (β=0.16, P=0.005) with a effect size (R square) 0.393 (this is a large effect size) , considering the precedently increased number of older adults world widely, and the few chanagable predictors available to reduce the cognitive disorders, our findings provide primary evidence that support comprehensive interventional program in need to maintain the cognitive function in community level and individual level.

Also, we have performed sensitivity analysis using the SEM, and the results validated the methodological robustness of mediation analysis (Fig 3). And there are statisticians suggested that the significant association of dependent variable with independent variable is unnecessary in the process of mediation analysis (Zhao et al, 2010).

Zhao X , Jr J G L , Chen Q . Reconsidering Baron and Kenny: Myths and Truths about Mediation Analysis[J]. Journal of Consumer Research, 2010, 37(2):197-206.

3. Comments of the reviewer are quite reassuring. Yet I feel that the question regarding how you adjusted the variables in the regression analysis is important (which in the form of quantitative, dichotomous, and categorical), to be sure that the assumptions of the regression model are fulfilled. Moreover, the analyses were adjusted with many variables which could lead to multicollinearity issue.

**Response:** In this study, we used the quantitative form of the variable. All descriptions can be seed in line158-169 of pages 8-9. Meanwhile, we conducted the collinearity diagnostics, and the results revealed no multicollinearity among those variables. As shown below, where the tolerance >0.1 and Variance Inflation Factor (VIF) <5 indicate multicollinearity can be ignored (Mason, 1991).

The collinearity diagnostics

|  | tolerance | VIF |  | tolerance | VIF |
| --- | --- | --- | --- | --- | --- |
| age | 0.927 | 1.078 | BMI | 0.975 | 1.026 |
| gender | 0.640 | 1.562 | smoking | 0.705 | 1.419 |
| residence | 0.702 | 1.424 | alcohol use | 0.793 | 1.262 |
| marital status | 0.936 | 1.069 | FPG | 0.987 | 1.013 |
| education | 0.619 | 1.615 | hyperlipidemia | 0.973 | 1.028 |
| ethnicity | 0.808 | 1.237 | hypertension | 0.953 | 1.050 |
| family income | 0.645 | 1.551 |  |  |  |

Dependent variable: MMSE score

4. The reviewer also suggests to use Structural Equation Modelling (SEM) approach to give a clearer picture of the overall pathway direction involving the variables and to validate the findings.

**Response:** We have performed the SEM analysis. The results as shown in Fig 3. The findings are consistent with the Bootstrap methods of PROCESS.

**Fig 3** The path analysis of the mediation effect of sleep duration in the relationship between social cohesion and MMSE

**Reviewers' comments:**
1. Page 8 Line 156-157, adding an hour to the sleep count for someone who took afternoon nap is less accurate without asking the subjects the number of hours.
**Response:** As any self-report measurement, the accuracy of data can not fully be guaranteed. According to our study, most people reported that their afternoon nap time was more or less 1 hour; statistically, it’s reasonable to adjust the total sleep hour by adding one hour to those who report having an afternoon nap.

2. Page 9 Line 169-170, proper citation for SPSS including publisher name to be stated.

**Response:** We revised the description of SPSS as “Analyses were performed using the Statistical Package for the Social Sciences (SPSS) version 24.0 (IBM Inc., Chicago, Illinois, USA)”.

3. Page 9 Line 165, the definition criteria for alcohol use is inaccurate and could have classified it in a day or in a week for the past 12 months.
**Response:** We have modified the definition as: a drink of at least one glass of alcohol, that equals 1/2 bottle of beer or 125-milliliter grape wine or fruit wine or 40-milliliter white wine, in a day for the past 12 months.

4. Page 9 Line 173, the sentence requires revision.

**Response:** We revised the sentence “A correlation matrix was created using partial correlations under controlling for age, gender, ethnicity, residence, educational attainment, marital status, family income, BMI, smoking, alcohol use, FBG, hyperlipidemia, hypertension” as “Partial correlations were employed to create correlation matrix under controlling for age, gender, ethnicity, residence, educational attainment, marital status, family income, BMI, smoking, alcohol use, FBG, hyperlipidemia, hypertension”.

5. Page 9 Line 177, for Hayes [28]was, was to be spaced out.
**Response:** we rephrased the sentence as “Bootstrap methods of PROCESS developed by Hayes was employed to test the mediation effect of sleep duration on the relationship between SC and MMSE score [29]”.

6. Page 10 Table 1, proper symbol for chi-square to be provided. The symbol chi-square and t and statistical tests which were employed in Table 1 to be stated in the statistical analysis section and denoted in the table footnote.
**Response:** We have symbolled the two different tests in table 1 and denoted the symbol of chi-square and t-tests in the Table 1 footnote.

7. Page 11 Table 2, an explanation or a note to be provided on how the variables other than continuous/dichotomous variables were adjusted in the partial correlation in the table footnote. The name of the correlation coefficient to be stated.
**Response:** We have added a footnote as “The covariates include age, gender, ethnicity, residence, education, marital status, family income, BMI, smoking, alcohol use, FBG, hyperlipidemia, hypertension were adjusted using Partial correlation method.”

8. Page 11 Line 197, r=-0.09 to be replaced with r=-0.11

**Response:** We have corrected this error.

9. Page 11 Line 200, what ‘multivariate’ refers to in Table 3 to be clearly denoted.

**Response:** We have clarified this issue, and denoted in the Table 3 where explained each of the models.

10. Page 12 Table 3, Model 2 and Model 3 in the table footnote to be written in a new line. Symbol β to be denoted in the table footnote. There were many variables with different type of data/scale of measurement other than continuous/dichotomous variables that were adjusted in the analysis. How these variables were coded and employed in the analysis to be clearly stated (Likewise with Table 4). A note on statistical assumptions fulfillment would be useful. Model summary to be provided.
**Response:** in Table 3, Model 2 and Model 3 in the table footnote have already been written in a new line and Symbol β was denoted in the table footnote. Additionally, variables other than continuous/dichotomous variables that were marital status and family income, we coded them in page 16 of measurement section. Model summary was provided in Statistical Analyses section as well as denoted in the table 3.

Three separate linear regression models were performed to examine the association among SC, sleep duration, and MMSE. In summary, model l include the SC and sleep duration; model 2 adjusted with covariate variables (age, gender, ethnicity, residence, education, marital status, family income, body mass index, smoking, alcohol use, fasting blood glucose, hyperlipidemia, hypertension); and the interaction between SC and sleep duration were added in model 3. Social interaction and social cohesion were tested separately in all the models.

11. Page 13 Line 216-219, Table 5, whether the analysis were adjusted or otherwise to be clearly stated.
**Response:** Yes, the analysis were adjusted covariate variables, and we supplemented the description “After controlling for age, gender, ethnicity, residence, education, marital status, family income, BMI, smoking, alcohol use, FBG, hyperlipidemia, hypertension.” In the table 5 footnote.

12. SEM approach could be explored or as a validation for the regression analysis.
**Response:** We peformed the SEM approach to validate the findings, can be seen in sensitivity analysis section and the results showed in Fig 3.

13. Not all references conformed to the journal format.

**Response:** We have formatted the reference list carefully; now it’s more suitable for the journal style.
