# Supplementary material for: Social capital and cognitive decline: Does sleep duration mediate the association?
Source: PLoS One. 2021 May 27;16(5):e0252208. doi: 10.1371/journal.pone.0252208 (PMC8158899; doi:10.1371/journal.pone.0252208)
Supplement: Attachment — Submitted filename: Response to Reviewers.docx [file pone.0252208.s004.docx]

Dear editor:

We are submitting the revised manuscript titled “Social capital and cognitive decline: does sleep duration mediate the association?” for publication. We have modified the manuscript along the lines suggested by editors and reviewers. In addition, we re-checked the reference and now it is complete and correct. All the changes are marked in red.

Below are our responses to the reviewer’s comments point by point.

1. Line 160, for marital status (unmarried, married and widowed/divorced), was the variable collapsed (0.1) for the regression analysis?

**Response:** We devided marital status into two categories (married vs. unmarried/widowed/divorced) in all the statistical analysis due to the few reponders are unmarried (10 of the 955). And we have running the analysis procedure again, all the changes marked in red.

2. Line 161- 161, it was mentioned that family income <1,000 RMB,162 1,000-1,999 RMB, 2,000-2,999 RMB, 3,000-4,999 RMB, and 5,000 RMB or more were collected. For Table 1, was the family income variable coded as <1000 and ≥ 1000 (0 and 1)? If so, which one was used in the regression analysis; (0.1) or scale data?

**Response:** The value of family income was ordinal variable in the regression model (divided into five degree: <1,000 RMB,162 1,000-1,999 RMB, 2,000-2,999 RMB, 3,000-4,999 RMB, and 5,000 RMB or more). We have also rehearched it in statistical analysis part as “family income (ordinal)”, marked in red.

3. Line 200, likewise for the partial correlation analysis, marital status and family income to be clearly denoted.

**Response:** As described above, We have revised the marital status and family income as marital status (married vs. not married (included unmarried and widowed/divorced)), family income (ordinal)”, now it is more clearly.
